# The Diabetes Pearl: Diabetes biobanking in The Netherlands

**DOI:** 10.1186/1471-2458-12-949

**Published:** 2012-11-06

**Authors:** Esther van’t Riet, Miranda T Schram, Evertine J Abbink, Wanda M Admiraal, Marja W Dijk-Schaap, Frits Holleman, Giel Nijpels, Behiye Özcan, Hanno Pijl, Nicolaas C Schaper, Eric JG Sijbrands, Bianca Silvius, Cees J Tack, Harold W de Valk, Bruce HR Wolffenbuttel, Coen DA Stehouwer, Jacqueline M Dekker

**Affiliations:** 1 VU University Medical Center, EMGO Institute for Health and Care Research, Amsterdam, The Netherlands; 2 VU University Medical Center, Department of Epidemiology and Biostatistics, Amsterdam, The Netherlands; 3 Maastricht University Medical Center, Department of Internal Medicine, Maastricht, The Netherlands; 4 Maastricht University Medical Center, Cardiovascular Research Institute Maastricht, Maastricht, The Netherlands; 5 Radboud University Nijmegen Medical Center, Department of Internal Medicine, Nijmegen, The Netherlands; 6 Academic Medical Center Amsterdam, Department of Internal Medicine, Amsterdam, The Netherlands; 7 Leiden University Medical Center, Department of Edocrinology & Metabolism, Leiden, The Netherlands; 8 VU University Medical Center, Department of General Practice, Amsterdam, The Netherlands; 9 Erasmus University Medical Center, Department of Internal Medicine, Rotterdam, The Netherlands; 10 Maastricht University Medical Center, Department of Endocrinology, Maastricht, The Netherlands; 11 Maastricht University Medical Center, School for Public Health and Primary Care, Maastricht, The Netherlands; 12 University Medical Center Utrecht, Department of Internal Medicine, Utrecht, The Netherlands; 13 University Medical Center Groningen, Department of Endocrinology, Groningen, The Netherlands

**Keywords:** Design, Biobank, Research infrastructure, Type 2 diabetes, The Netherlands

## Abstract

**Background:**

Type 2 diabetes is associated with considerable comorbidity and severe complications, which reduce quality of life of the patients and require high levels of healthcare. The Diabetes Pearl is a large cohort of patients diagnosed with type 2 diabetes, covering different geographical areas in the Netherlands. The aim of the study is to create a research infrastructure that will allow the study of risk factors, including biomarkers and genetic determinants for severe diabetes complications.

**Methods/design:**

Baseline examinations began November 2009 and will continue through 2012. By the end of 2012, it is expected that 7000 patients with type 2 diabetes will be included in the Diabetes Pearl cohort. To ensure quality of the data collected, standard operation procedures were developed and used in all 8 recruitment centers. From all patients who provide informed consent, the following information is collected: personal information, medication use, physical examination (antropometry, blood pressure, electrocardiography (ECG), retina photographs, ankle-brachial index, peripheral vibration perception), self-report questionnaire (socio-economic status, lifestyle, (family) history of disease, and psychosocial well-being), laboratory measurements (glucose, A1c, lipid profile, kidney function), biobank material (storage of urine and blood samples and isolated DNA). All gathered clinical data and biobank information is uploaded to a database for storage on a national level. Biobanks are maintained locally at all recruitment centers.

**Discussion:**

The Diabetes Pearl is large-scale cohort of type 2 diabetes patients in the Netherlands aiming to study risk factors, including biomarkers and genetic markers, for disease deterioration and the development of severe diabetes complications. As a result of the well-designed research design and the national coverage, the Diabetes Pearl data can be of great value to national and international researchers with an interest in diabetes related research.

## Background

### The string of pearls initiative

The String of Pearls Initiative is the product of a unique partnership between all eight university medical centres (academic teaching hospitals) in the Netherlands, established in 2007 by the NFU (Netherlands Federation of University Medical Centres). The main aim of the String of Pearls Initiative is to create a national research infrastructure, in which biobanks are established in patient cohorts, combined with a uniform and central clinical database.

Initially, eight patient cohorts were created, the so-called “pearls”: cerebrovascular accident, type 2 diabetes mellitus, hereditary colorectal caner, inflammatory bowel diseases (Crohn’s disease and ulcerative colitis), leukaemia, neurodegenerative diseases, renal failure, rheumatoid arthritis and arthrosis. In a standardised way, patient materials and patient data are contributed to each of the patient cohorts in each of the Dutch University Medical Centers [1].

### Type 2 diabetes

The International Diabetes Federation estimated that in 2011 approximately 366 million people worldwide had type 2 diabetes, and they expect this number to have risen to 552 million in 2030 [2]. An increase in the prevalence of type 2 diabetes is expected to be a result of several trends: aging of the population, increased life-expectancy of patients with type 2 diabetes, earlier detection of type 2 diabetes and increases in the prevalence of the main risk factors for type 2 diabetes, i.e. adiposity and lack of physical activity.

Diabetes is associated with considerable comorbidity and severe complications: cardiovascular disease, nephropathy, retinopathy and neuropathy. These complications reduce quality of life of the patient and are a huge burden for national healthcare. Compared to the general non-diabetic population, patients with type 2 diabetes have a 2 to 4 fold higher risk of death from CVD [3]. There is, however, considerable variation in the risk of diabetes complications within the diabetes population. A large proportion of patients respond quite well to current therapies. Only a relatively small proportion (but still a large numbers of patients due to the high prevalence of diabetes) cannot be controlled adequately, and have a very high risk of developing severe complications [4]. At present, no markers are available to predict the effectiveness of therapies, or to identify vulnerable patients who are at high risk of diabetes complications.

Patients with type 2 diabetes in the Netherlands mainly receive care from their general practitioner (primary care). In case of complications or inability to achieve glycemic control, the patient is referred to secondary care, e.g. specialists in internal medicine, endocrinology, ophthalmology and cardiology. In very complex cases in which high specialist care is needed, patients are referred to an academic medical center (tertiary care). In 2008, it was estimated that the number of diabetes related comorbidities was twice as high in secondary care compared to primary care. Thus, the combination of patients treated in primary, secondary and tertiary care, is of utmost importance for generating a representative overview of type 2 diabetes and type 2 diabetes complications in the Netherlands.

### Objective of the Diabetes Pearl

The Diabetes Pearls is a large cohort of patients diagnosed with type 2 diabetes, covering different geographical areas in the Netherlands and covering patients treated in primary, secondary and tertiary care. The aim of the study is to create a research infrastructure that will allow the study of (new) risk factors, including biomarkers and genetic determinants for severe diabetes complications. The overarching goal is to develop new, more targeted and effective therapies that will improve diabetes care and reduce the burden of diabetes.

## Methods/Design

### Study design

The Diabetes Pearl is an observational cohort study, in which all eight Dutch academic medical centers participate. The initial organization of the String of Pearls Initiative in the Netherland was cofinanced by the Dutch Government and the eight Dutch academic medical centers. The continuation is financed by the academic medical centers.

### Study population and inclusion criteria

**Figure 1:**
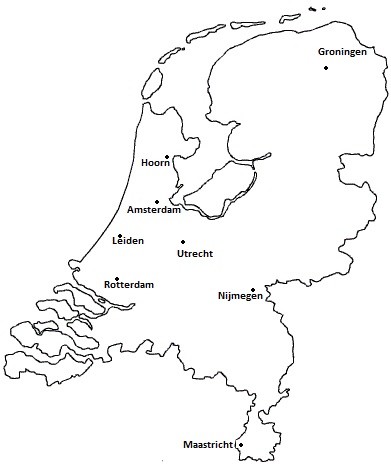
Geographical location of the inclusion centers of the Diabetes Pearl in the Netherlands.

Patients who were diagnosed with type 2 and who received secondary or tertiary medical care in one of the 6 academic medical centers in Amsterdam, Utrecht, Nijmegen, Rotterdam, Leiden or Groningen, primary medical care in the area of Hoorn or one of the three types of care in the region of Maastricht, were eligible for participation in the Diabetes Pearl (Figure 1). Patients were excluded if their ability to understand and write the Dutch language enabled them to provide written informed consent.

### Ethical considerations, patient information and written informed consent

All protocols used to create the Diabetes Pearl, and all generic String of Pearl biobanking and data-procedures were reviewed as a multi-center study by the ethics committee of the VU University Medical Center. Ethical Committies of all seven other academic centers provided positive advise for participation. To provide patients with enough time to decide whether or not they wanted to participate, study information including an informed consent form was sent by mail prior to their clinic visit. Upon arrival at the clinic, an oral explanation was provided and patients who agreed to participate, signed the informed consent form. Written informed consent included participation in the study itself as well as separate consent items covering biobanking, genetic analysis, and linkageto important national databases like the municipality register for mortality follow-up. All participants received a copy of the signed informed consent.

### Baseline data collection of the Diabetes Pearl

Since the start of the String of Pearls Initiative in 2007, all recruitment centers have worked together to create the optimal dataset needed to gather baseline data of patients with type 2 diabetes in the Netherlands. This minimal dataset was supported by local coordinators of the participating clinics and introduced accordingly. In addition, work instructions were translated into standard operational procedures (SOPs) to ensure comparability of all data collected.

Baseline examinations began November 2009 and will continue through 2012. From all patients who provide informed consent, the following information was collected:

1. Demographics and medication use

Hospital information systems at all recruitment centers were used to collect information on date of birth and gender. Participants were requested to bring all their medication on the day of the visit or lists from pharmacists were used so that medication use (name, indication for description, dose, frequency, ATC-code) could be reported accurately.

2. Questionnaire data

All participants received a self-report questionnaire in which the following items were included: socioeconomic status of the participants and their partner, smoking behavior, alcohol consumption habits and (family) history of diabetes, stroke and CVD. In addition, a modified version of the Rose Questionnaire for the diagnosis of ischaemic heart pain and intermittent claudication [5] and the EuroQol 5D questionnaire for information on quality of life [6] were included. In 7 of the 8 recruitment centers, the questionnaire was extended with the following items: information on hypoglycaemic events, the SF-12 questionnaire on quality of life [7], the PAID Questionnaire on diabetes-related emotional distress [8], and the DN4 questionnaire on neuropathic pain [9].

3. Physical examinations

Weight and height were measured bare foot and wearing light clothing using a clinical stadiometer and scale. Waist circumference was measured between the lower rib margin and the spina iliaca anterior superior, and the hip circumference over the maximum of the buttocks. A tape measure was used in all recruitment centers. Two measurements of both waist and hip were performed. Final waist and hip circumference were calculated as the mean of the two measurements. Blood pressure was determined three times on the right arm after a 10 minute rest period, using a non-invasive blood pressure monitor (Omron 7051 T in seven centers, Colin Press BP 8800p in one center). Final blood pressure was calculated as the mean of the last 2 measurements.

4. Electrocardiogram

A rest 12-lead electrocardiogram (ECG) was performed on all participants. ECGs were archived electronically.

5. Fundus photography

To determine the presence of diabetic retinopathy, fundus photography of both eyes (with dilated pupils in case no clear picture could be obtained) was performed in 7 of the 8 clinics. All fundus photographs were performed with a non-mydriatic camera in 45 degrees of at least two fields: one field centered on the macula and one nasal field with the optic disc positioned on a disc-diameter from the temporal edge of the field. This is in accordance with the EURODIAB protocol [10].

6. Ankle-brachial blood pressure index

As indicator of peripheral arterial disease, we obtained an ankle-brachial blood pressure index from all participants in 7 out of the 8 centers. Blood pressures were measured at the brachial artery of the left and right arm, and twice at the dorsal pedal artery and posterior tibial artery of the left and right ankle. The ankle-brachial index was calculated for every ankle separately as the highest recorded pressure on that ankle divided by the highest pressure in the brachial artery. In 6 centers, the blood pressures were determined with a Doppler device and the index was calculated by hand. In one center, the Omron VP2000 was used, which determined the blood pressures automatically and calculated the corresponding ankle-brachial index.

7. Peripheral vibration perception

With the use of a Horwell Neurothesiometer (Scientific Laboraty Supplies, Nottingham, UK), peripheral vibration threshold was tested in 7 centers. Vibration thresholds were tested 8 times at the distal phalanx of the hallux. Mean vibration threshold was then calculated as the mean of the 6 highest vibration thresholds.

8. Laboratory measurements

Fasting venous blood plasma was used to determine fasting glucose, total cholesterol, HDL cholesterol, triglycerides and serum creatinin. A fasting whole blood sample was used for the determination of HbA1c level. In addition, a sample of morning urine was collected and used for the determination of urine albumin and urine creatinin. All the laboratories were certified and located on site in the 8 clinics.

9. Biobanking

An important part of the Diabetes Pearl is creating a national biobank. Therefore, additional samples of venous blood (serum, EDTA plasma, citrate plasma) and morning urine (including and excluding anti-oxidants) were collected for the storage of plasma, serum and urine. Samples were aliquoted and stored at -80 degrees. In addition, an EDTA whole blood sample was collected for DNA extraction. After isolation (on site in all 8 recruitement centers), a quality control on the DNA was performed and DNA was catalogued and stored in -80 degrees Celsius storage freezers.

### Data processing

Since the start of the Diabetes Pearl, much attention is given to create a solid research infrastructure. As a result, the Diabetes Pearl consists of a well-designed minimal dataset and the use of standardized data collection procedures across centres. In addition, clinical data, questionnaire data, laboratory data and biobank information are stored on a national level in a highly sophisticated data system. Before the data are uploaded nationally, several data checks are performed to investigate completeness and correctness of the data. Moreover, participant’s name and address is kept only at the recruitment center, all uploaded data are pseudonomized. Biobank material is also stored anonymous and standardized storage guidelines have been developed. Biobank storage is provided local at all recruitement centers.

### Preliminary results

Until February 2012, 4.587 patients were included in the Diabetes Pearl of whom 2.320 received primary medical care and 2.267 received secondary or tertiary medical care. It is to be expected that by the end of 2012, a total of 7000 patients will be included.

## Discussion

The Diabetes Pearl is a national joined initiative of all University Medical Centers in the Netherlands, aiming to create a research infrastructure that will allow the study of (new) risk factors for disease deterioration and the development of severe diabetes complications. The overarching goal is to develop new, more targeted and effective therapies, which will improve diabetes care and reduce the burden of diabetes.

### The value of the current Diabetes Pearl cohort

Internationally, the use of patient data is increasing in scientific research. Patient cohort data have the advantage of inclusion of large numbers of patients over wide coverage. Due to the resemblance with usual care, patient cohort data can be of great importance in scientific research and the production of clinical care guidelines [11]. For example, the General Practitioners Research Database (GPRD) in the UK, the Swedish National Diabetes Register and the China Kandoori Biobank, have been valuable in various research topics concerning diabetes [12-15]. Recently, members of our research group have used patient data from the Diabetes Care System West-Friesland to report on the course of retinopathy in type 2 diabetes patients receiving structured primary care in the Netherlands [4].

The strengths of the Diabetes Pearl over other (national and international) patient cohorts include:1) the national coverage due to the collaboration between the 8 academic medical centers in the Netherlands, 2) the inclusion of patients receiving primary care as well as secondary and tertiary care, providing a representative overview of type 2 diabetes patients, 3) the combination of clinical data with the collection of biomaterials, enabling future studies to include determination of specific biomarkers, including genetic and metabolic markers, in relation to the occurrence of diabetes complications, 4) the profound phenotyping, providing essential diabetes-specific information not available in usual diabetes care nor in general population biobanking cohorts, 5) the well-designed research infrastructure, enabling the use of the Diabetes Pearl biobank and database by other researchers, and 6) the use of standardizes protocols throughout all recruitment centers. These specific features create a unique research infrastructure, which promises to be of great value in gaining more insight in the pathogenesis of type 2 diabetes complications.

The Diabetes Pearl cohort entirely exists of type 2 diabetes patients, which is in concordance with our main aim to study diabetes complications. To study gene-environment interaction in for example the development of type 2 diabetes, a representative set of cases and controls is needed. For example, the InterAct Consortium has established a large-scale case–control study, aiming to investigate the influence of genetic and lifestyle factors on the risk of type 2 diabetes. To open the possibility to include healthy controls in our studies, all standard operational procedures were harmonized with large population studies in the Netherlands, like the Hoorn Study [16] the Maastricht Study and the LifeLines cohort.

### Follow-up

The ultimate goal of the Diabetes Pearl Research Group is to increase the knowledge of the occurrence, prevention and effective treatment of diabetes complications. To reach this goal, we need more insight into the occurrence and the determinants and course of diabetes complications, and we need to be able to perform research on new, targeted treatment strategies. Therefore, we aim to include at least one follow-up measurement. All baseline measurements will then be repeated to be able to investigate changes and prospective associations. In addition, we have started procedures to initiate a continuous registration of morbidity and mortality events in all participants. With the help of the national municipality register and medical files from local hospitals, accurate information on survival status as well as all morbidity events will be available. Moreover, initiatives to link the Diabetes Pearl to a national database with accurate information on medication use, have started. The resulting longitudinal investigation of type 2 diabetes patients can be used in for example the identification of risk factors for diabetes complications, the identification of individuals at high-risk of complications and the identification of effective treatment strategies to prevent diabetes complications.

## Conclusion

In conclusion, this paper described the design of the Diabetes Pearl, a large-scale, clinically well-defined cohort of type 2 diabetes patients in the Netherlands aiming to study risk factors, including biomarkers and genetic markers, for disease deterioration and the development of severe diabetes complications. As a result of this well-designed study, the national coverage and the inclusion of patients in all stages of the disease, the Diabetes Pearl data can be of great value to national and international researchers to unravel the pathophysiology of type 2 diabetes.

## Competing interests

The author(s) declare that they have no competing interests.

## Authors’ contributions

ER: study design, data collection, local study coordination, wrote manuscript. MS: study design, data collection, local study coordination, critically reviewed and approved manuscript. EA: study design, data collection, local study coordination, critically reviewed and approved manuscript. WA: data collection, local study coordination, critically reviewed and approved manuscript. MDS: data collection, critically reviewed and approved manuscript. FH: local study coordination, critically reviewed and approved manuscript. GN: data collection, local study coordination, critically reviewed and approved manuscript. BÖ: study design, data collection, local study coordination, critically reviewed and approved manuscript. HP: study design, local study coordination, critically reviewed and approved manuscript. NS: study design, local study coordination, critically reviewed and approved manuscript. ES: study design, local study coordination, critically reviewed and approved manuscript. BS: data collection, critically reviewed and approved manuscript. CT: study design, local study coordination, critically reviewed and approved manuscript. HV: study design, local study coordination, critically reviewed and approved manuscript. BW: study design, local study coordination, critically reviewed and approved manuscript. CS: initiated study, local study coordination, national study coordination, critically reviewed and approved manuscript. JD: initiated study, local study coordination, national study coordination, critically reviewed and approved manuscript.

## Pre-publication history

The pre-publication history for this paper can be accessed here:

http://www.biomedcentral.com/1471-2458/12/949/prepub
